# Can Clinical and Surgical Parameters Be Combined to Predict How Long It Will Take a Tibia Fracture to Heal? A Prospective Multicentre Observational Study: The FRACTING Study

**DOI:** 10.1155/2018/1809091

**Published:** 2018-04-30

**Authors:** Leo Massari, Francesco Benazzo, Francesco Falez, Ruggero Cadossi, Dario Perugia, Luca Pietrogrande, Domenico Costantino Aloj, Antonio Capone, Michele D'Arienzo, Matteo Cadossi, Vincenzo Lorusso, Gaetano Caruso, Matteo Ghiara, Luigi Ciolli, Filippo La Cava, Marco Guidi, Filippo Castoldi, Giuseppe Marongiu, Alessandra La Gattuta, Dario Dell'Omo, Michelangelo Scaglione, Sandro Giannini, Mattia Fortina, Alberto Riva, Pier Luigi De Palma, Antonio Pompilio Gigante, Biagio Moretti, Giuseppe Solarino, Francesco Lijoi, Giovanni Giordano, Pier Giorgio Londini, Danilo Castellano, Giuseppe Sessa, Luciano Costarella, Antonio Barile, Mariano Borrelli, Attilio Rota, Raffaele Fontana, Alberto Momoli, Andrea Micaglio, Guido Bassi, Rossano Stefano Cornacchia, Claudio Castelli, Michele Giudici, Mauro Monesi, Luigi Branca Vergano, Pietro Maniscalco, M'Putu Bulabula, Vincenzo Zottola, Auro Caraffa, Pierluigi Antinolfi, Fabio Catani, Claudio Severino, Enrico Castaman, Carmelo Scialabba, Venceslao Tovaglia, Pietro Corsi, Paolo Friemel, Marco Ranellucci, Vincenzo Caiaffa, Giovanni Maraglino, Roberto Rossi, Antonio Pastrone, Patrizio Caldora, Claudio Cusumano, Pier Bruno Squarzina, Ugo Baschieri, Ettore Demattè, Stefano Gherardi, Carlo De Roberto, Alberto Belluati, Antonio Giannini, Ciro Villani, Pietro Persiani, Silvio Demitri, Bruno Di Maggio, Guglielmo Abate, Francesca De Terlizzi, Stefania Setti

**Affiliations:** ^1^Orthopaedic and Traumatology Department, “S. Anna” Hospital, University of Ferrara, Ferrara, Italy; ^2^Orthopaedic and Traumatology Department, IRCCS Foundation “San Matteo” Hospital, University of Pavia, Pavia, Italy; ^3^Orthopaedic and Traumatology Department, “Santo Spirito in Sassia” Hospital, Rome, Italy; ^4^Research and Development, IGEA Clinical Biophysics, Carpi, Modena, Italy; ^5^Orthopaedic and Traumatology Department, “Sant'Andrea” Hospital, Rome, Italy; ^6^Health Sciences Department, Operative Unit of Orthopaedics and Traumatology, “San Paolo” Hospital, University of Milan, Milan, Italy; ^7^Orthopaedic, Traumatology and Rehabilitation Department, II Orthopaedics Clinic, CTO Hospital, Torino, Italy; ^8^Orthopaedic Department, University of Cagliari, Cagliari, Italy; ^9^Orthopaedic and Traumatology Department, “Paolo Giaccone” Hospital, University of Palermo, Palermo, Italy; ^10^Department of Orthopaedic Surgery, Rizzoli Orthopaedic Institute, University of Bologna, Bologna, Italy; ^11^Translational Research on New Surgical and Medical Technologies Department, Orthopaedics and Traumatology II°, University of Pisa, Pisa, Italy; ^12^Orthopaedics and Traumatology Clinic, “S. M. alle Scotte” Hospital, University of Siena, Siena, Italy; ^13^Clinical and Molecular Science Department, Faculty of Medicine, Polytechnic University of Marche, Ancona, Italy; ^14^Basic Medical Science, Neurosciences and Sensory Organs Department, University of Bari, Bari, Italy; ^15^Orthopaedic and Trauma Department, “Morgagni-Pierantoni” Hospital, Forlì, Italy; ^16^Orthopaedic and Traumatology Department, “Misericordia” Hospital ASL 9, Grosseto, Italy; ^17^Surgery Department, “Vittorio Emanuele” Hospital, University of Catania, Catania, Italy; ^18^Orthopaedic and Trauma Department, “San Michele” Nursing Home Hospital, Maddaloni, Caserta, Italy; ^19^Orthopaedic and Traumatology Department, “Sandro Pertini” Hospital, ASL RMB, Rome, Italy; ^20^Orthopaedic and Traumatology Department, “San Bortolo” Hospital, Vicenza, Italy; ^21^Orthopaedic and Traumatology Department, A.O. Pavia Voghera Hospital, Pavia, Italy; ^22^Orthopaedic and Traumatology Unit, Ariano Irpino Hospital, Avellino, Italy; ^23^Orthopaedics and Trauma Department, “Papa Giovanni XXIII” Hospital, Bergamo, Italy; ^24^Orthopaedic and Traumatology Department, “M. Bufalini” Hospital, Cesena, Italy; ^25^Orthopaedic and Traumatology Department, “Guglielmo da Saliceto” Hospital, Piacenza, Italy; ^26^Traumatology and Reconstructive Surgery Functional Department, “S. Anna” Hospital, Como, Italy; ^27^Orthopaedics and Traumatology Clinic, “S. M. Misericordia” Hospital, University of Perugia, Perugia, Italy; ^28^Orthopaedic Surgery Department, Policlinico di Modena, University of Modena and Reggio Emilia, Modena, Italy; ^29^Orthopaedic and Traumatology Department, Montecchio Maggiore Hospital, Vicenza, Italy; ^30^Orthopaedic and Traumatology Department, CTO Hospital ASL RM “C”, Rome, Italy; ^31^Orthopaedic and Traumatology Department, Regione Veneto Azienda ULSS 18, Rovigo, Italy; ^32^Orthopaedics and Traumatology Department, “Di Venere” Hospital, Bari, Italy; ^33^Orthopaedics and Traumatology Department, “SS. Annunziata” Hospital, Taranto, Italy; ^34^Orthopaedic and Traumatology SCDU Department, “Mauriziano Umberto I” Hospital, University of Torino, Torino, Italy; ^35^Orthopaedic and Traumatology Surgery Department, “San Donato” Hospital, Arezzo, Italy; ^36^Orthopaedics Department, NOCSAE Hospital, Modena, Italy; ^37^Orthopaedics and Traumatology Department, “Santa Chiara” Hospital, Trento, Italy; ^38^Orthopaedics Unit, “Santa Maria di Loreto Mare” Hospital, Loreto Mare, Napoli, Italy; ^39^Specialized Surgery Department, “S. Maria delle Croci” Hospital, Ravenna, Italy; ^40^Orthopaedic Department, Sapienza University of Rome, Rome, Italy; ^41^Orthopaedic and Trauma Department, “Santa Maria della Misericordia” Hospital, AOUD Udine, Udine, Italy; ^42^Orthopaedics and Traumatology Unit, Piedimonte Matese Hospital, Caserta, Italy

## Abstract

**Background:**

Healing of tibia fractures occurs over a wide time range of months, with a number of risk factors contributing to prolonged healing. In this prospective, multicentre, observational study, we investigated the capability of FRACTING (tibia FRACTure prediction healING days) score, calculated soon after tibia fracture treatment, to predict healing time.

**Methods:**

The study included 363 patients. Information on patient health, fracture morphology, and surgical treatment adopted were combined to calculate the FRACTING score. Fractures were considered healed when the patient was able to fully weight-bear without pain.

**Results:**

319 fractures (88%) healed within 12 months from treatment. Forty-four fractures healed after 12 months or underwent a second surgery. FRACTING score positively correlated with days to healing: *r* = 0.63 (*p* < 0.0001). Average score value was 7.3 ± 2.5; ROC analysis showed strong reliability of the score in separating patients healing before versus after 6 months: AUC = 0.823.

**Conclusions:**

This study shows that the FRACTING score can be employed both to predict months needed for fracture healing and to identify immediately after treatment patients at risk of prolonged healing. In patients with high score values, new pharmacological and nonpharmacological treatments to enhance osteogenesis could be tested selectively, which may finally result in reduced disability time and health cost savings.

## 1. Introduction

Over the past 50 years, orthopaedic surgery has defined, for the different skeletal sites and different fracture morphologies, guidelines to ensure a suitable mechanical environment to allow healing [1]. The treatment of tibia fractures has become almost solely surgical, using nails, plates, and screws and external fixation, to set the mechanical conditions (stability, contact, and alignment of the fracture fragments) for bone repair [2–4]. Following surgical treatment, patients have obtained significant benefits; limb function recovery is more rapid, and joint stiffness or local osteoporosis is rare.

Bone healing results from the activity of different cell populations at the fracture site [5, 6]; nowadays, orthopaedic research aims to stimulate bone callus formation by pharmacological [7], cellular [8], and biophysical means [9] in order to speed up fracture healing [10].

The tibia is the region with the highest incidence of fractures resulting from trauma [3]. The healing of a tibia fracture can occur over a very wide time range, from a minimum of 2 months to a maximum of 6 months in most patients. Nevertheless, in a significant percentage of patients, healing may take place well beyond 6 months after the trauma or may require one or more surgical procedures, with significant associated health costs [11–13].

Although general and local conditions that may adversely affect fracture healing have been identified [14–18], the ability to early recognise fractures at risk of developing a nonunion is still left to the surgeon's experience.

In a previous retrospective study, we assessed clinical records of patients treated for tibia fractures to collect information on trauma characteristics, fracture treatment, patient's general conditions, and finally the time required for fracture healing. The data thus collected were analysed by logistic regression to identify those parameters that influenced time to fracture healing, and then they were combined in a score whose values increased as time to healing increased [19].

We conducted this prospective multicentre observational study to (i) investigate, in a large cohort of patients, if the score, calculated immediately after the treatment, could reliably predict the time to healing of a tibia fracture and (ii) determine the ability of the score to identify fractures at risk of nonunion, that is, healing after more than 6 months.

## 2. Materials and Methods

This prospective observational study mirrors the clinical practice for tibia fracture treatment throughout the country, “real world data.” On this assumption, neither indication was given on how to treat the fracture nor the review on treatment appropriateness was performed.

From January 2010 to September 2012, patients who had suffered a tibia fracture were recruited in 41 Italian orthopaedic traumatology centres. Patient treatment was left to the choice of the trauma surgeon based on experience. All patients provided written, informed consent for the handling of personal data. The study was approved by the ethical committee of the coordinating centre: University of Ferrara, Italy.

Inclusion criteria were as follows: patients with posttraumatic fractures type 41-A and B, 42-A-B and C, 43-A and B according to AO classification [1]; fracture treatment within 3 days from trauma; and patient age > 18 years.

Exclusion criteria were as follows: fractures involving the tibia plateau and malleolar fractures, patients with autoimmune diseases or neoplasia, and patients who could not return to the treating centre for follow-up visits.

We selected a patient-centred end point to determine fracture healing: fully weight-bearing without pain. Within 12 months from trauma, the date at which the fracture healed was used to calculate days and months elapsed since treatment (“healing time”). We chose to follow patient for 12 months after treatment as a small percentage of fractures may slowly heal 6 months after the trauma [20–22].

Criteria for failure to heal included fractures not healed within 12 months from trauma and fractures that required surgical procedures not foreseen in the initial treatment plan.

### 2.1. Database

For each patient, surgical and clinical data were collected in dedicated software and used to calculate the score: FRACTING (FRACTure healING). Drop-down menu was used for descriptive variables. Required fields ensured complete and consistent data collection. The score was calculated adding all values shown in Table 1.

As ancillary information, on the day of healing, surgeons were asked to record on the database the presence of bone callus on tibia cortices in orthogonal radiographs; nevertheless no centralised review of the X-rays was performed.

### 2.2. Statistical Analysis

We conducted a power analysis to establish the number of patients required to demonstrate the correlation of the score with time to healing with a confidence interval of 0.10. Considering the correlation *r* = 0.69 observed in the retrospective study, we calculated a sample size of 301 patients.

In the descriptive analysis for continuous variables, mean values and standard deviations are reported. ANOVA analysis with post hoc Bonferroni test has been applied for comparison between multiple groups. The association between continuous variables was calculated by linear regression analysis and Pearson linear correlation coefficient. In order to determine the ability of the score to identify those who would not heal within 6 months posttrauma, contingency tests and receiver operating characteristic (ROC) analysis were used; specificity, sensitivity and positive predictive values were calculated. Data were analysed using SPSS 21.0 software [IBM, New York, USA].

## 3. Results

### 3.1. Study Cohort

**Figure 1:**
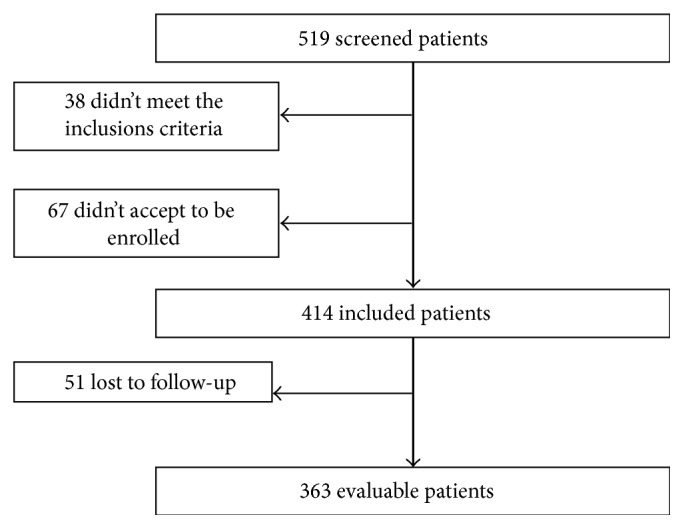
Flow diagram in which the eligible, screened, and included patients are illustrated.

519 patients were screened, 38 did not meet the inclusion criteria, and 67 did not accept to be enrolled. Finally, 414 patients with tibia fracture entered into the database. Fifty-one patients (12%) did not return for follow-up visits. Overall 363 patients completed the study (Table 2) (Figure 1).

Twenty-one percent of fractures were open; in 3%, loss of bone tissue occurred. In 75% of fractures, both tibia and fibula were fractured. According to AO classification, 6% of fractures were type 41, 72% were type 42, and 22% were type 43.  Table 3 reports the treatment performed in open or closed fractures.

**Figure 2:**
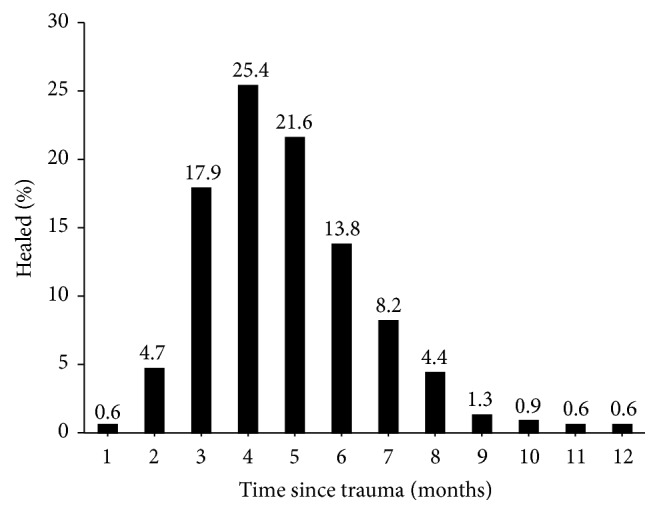
Percentage of fractures healed each month after treatment.

Out of 363 patients, 319 (88%) healed within 12 months; 268 (74%) healed within 6 months and 51 (14%) between 6 and 12 months. Forty-four fractures (12%) were considered failure as they required either further surgery or more than 12 months to heal.  Figure 2 shows the percentage of fractures healed at each month.

Fracture healing was achieved on average in 130 ± 54 days for all patients.

At healing, the presence of callus was reported for 311 fractures: in at least three cortices in 81% of patients, in two cortices in 15% of patients, and in one cortex only in 4% of patients.

At long-term follow-up (6 months from healing), 93% of fractures were reevaluated and their healing was confirmed; 2 patients reported that the fracture, initially judged to be healed, had over time been treated again.

### 3.2. Healing Time and FRACTING Score

The values of the score ranged from 3 to 18, with a mean value of 7.3 ± 2.5, median 7.

**Figure 3:**
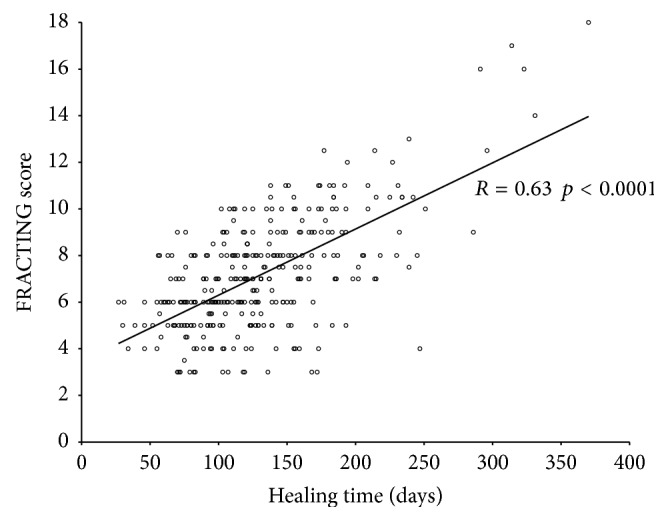
Linear correlation of the score value with fracture healing time in days.

The correlation of the score with healing time expressed in days is significant: *r* = 0.63; *p* < 0.0001 (Figure 3).

In traumatology practice, the patient follow-up interval after treatment is usually 30 days. Therefore, we grouped the fracture healing into five time intervals: ≤3, 4, 5, 6, and >6 months. The average score values by months after treatment are reported in Table 4.

**Figure 4:**
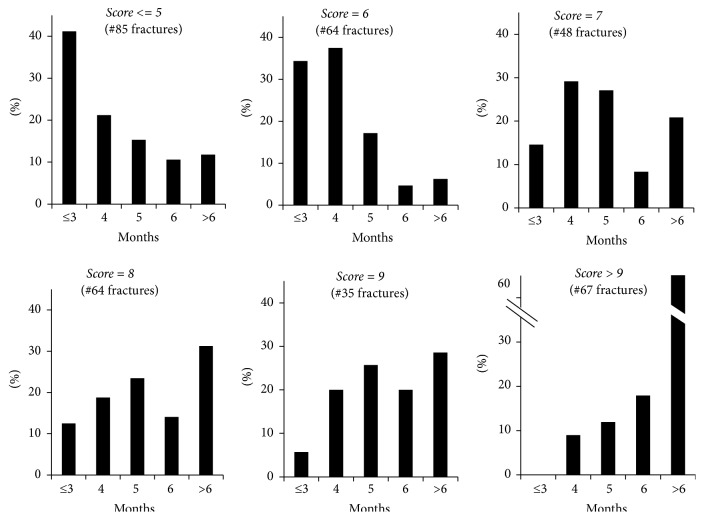
Healing time of fractures grouped by score values.

In further analysis we evaluated for different score values the percentage of fractures healed at different time intervals from trauma (Figure 4).

Among the 363 fractures, 12% of fractures with score values ≤ 7 took more than 6 months to heal versus 43% of fractures with score values > 7 (*p* < 0.0001). We performed the ROC analysis to evaluate the ability of the score to predict fracture healing in more than 6 months (nonunion); the area under the curve (AUC) was 0.823 ± 0.033 (*p* < 0.0001). Data for the sensitivity, specificity, and positive predictive value for individual score values are shown in Table 5.

## 4. Discussion

To estimate healing time of a fracture immediately after its treatment is difficult, it is based on individual surgeon's experience, and it is made even more difficult as to date there is no accepted gold standard to determine the healing of a fracture [23, 24]. Several clinical studies have considered healing on both (i) radiographic criteria: presence of bone callus in at least three cortices on radiographs performed in the two projections, and (ii) clinical criteria: absence of tenderness at the fracture site, the absence of pain on application of pressure to the fracture site and during full weight-bearing [21, 22, 25].

In this observational study, we tested the ability of the FRACTING score to estimate immediately after fracture treatment how long it will take to heal. Here, fracture healing has been based exclusively on clinical criteria: full weight-bearing without pain. This patient-centred end point is relevant in clinical practice as it corresponds to the return to work and daily activities. As confirmation of reliability of the criteria for healing adopted, at 6-month follow-up, in 2 patients only, further treatment was required.

The FRACTING score is positively correlated with the healing time in days (*r* = 0.63; *p* < 0.0001). Furthermore, ANOVA test shows a significant association among score values and healing time in months (Table 4).

Within each score value (Figure 4), we observed fractures healing at different time periods, thus leaving a range of uncertainty that can be explained by individual biology, patient's behaviour, and adherence to the orthopaedic surgeon indications until healing. Nevertheless, it is noteworthy that while for scores ≤5, 12% of fractures healed after 6 months from trauma, for scores >9, the percentage increased to 61% (*p* < 0.0001).

The ROC analysis shows good reliability of the FRACTING score to assess the risk of nonunion (AUC = 0.823). In clinical practice, an effective threshold might be selected for a score value of 8 that shows a sensitivity of 63% with specificity of 81%, and a positive predictive value of 53% that shows that the fracture heals in more than 6 months (Table 3).

To our knowledge this is the first attempt to prospectively validate a score to predict fracture healing time. The results of this study cannot be extended to skeletal segments other than the tibia. However, our work suggests that the same approach can be adopted to develop specific scores for fractures located in different bones.

The major strength stems from the population studied that represents real world data. FRACTING score is associated with fracture healing time and able to accurately identify fractures at risk of nonunion.

Limitations include the exclusive use of clinical criteria for definition of fracture healing, although only 2 patients experienced fracture retreatment at follow-up.

## 5. Conclusions

FRACTING score might be used for selecting patients in whom the efficacy of therapeutic interventions to enhance fracture healing is assessed, such as cell therapy, growth factors, drugs, or physical stimuli. Furthermore, patients with high scores may benefit from customised treatment protocols by planning closer surveillance and specific rehabilitation that might limit the occurrence of nonunions, thus leading to significant cost savings [12, 13].

## Figures and Tables

**Table 1:** Parameters used for FRACTING score calculation.

Parameter	Values for score calculation	
Age increase	18–45	1
	46–60	2
	>60	3

Malnutrition	Yes	1

Diabetes	Yes	1

Smoking	Yes	1

Use of NSAIDs	Yes	1

Fracture exposure severity	Closed	1
	Open grade 1	2
	Open skin < 5 cm	3
	Open skin > 5 cm	4

Location: metaphysis or epiphysis	Yes	1

Synthesis device	Nail	1
	Plate	2
	External fixation	3

Unstable	Yes	1

Misalignment > 5°	Yes	1

Bone graft	Yes	1

Plate + diastasis	Yes	0.5

Angular stability plate	Yes	0.5

Plate + plaster cast	Yes	−0.5

Fracture of tibia alone	Yes	1

Loss of bone substance	Yes	1

Bone diastasis, >2 mm	Yes	1

Length of surgery, >120 minutes	Yes	1

Blood haemoglobin before treatment < 10 g/dl	Yes	1

Blood haemoglobin after treatment < 10 g/dl	Yes	1

NSAIDs: nonsteroidal anti-inflammatory drugs.

**Table 2:** Patients' characteristics.

Male/female	257/106
Age (yrs)	48 ± 17
Weight (Kg)	74 ± 13
Height (cm)	171 ± 8

**Table 3:** Treatment performed in open or closed fractures.

	No. of fractures treated	Open	Closed
External fixation	76	40	36
Nail	163	25	138
Plate & screws	124	12	112

Total	363	77 (21%)	286 (79%)

**Table 4:** Average score values in different fracture healing months.

Healing months	≤3	4	5	6	>6
No. of fractures	74	81	69	44	51
Avg score (st.dev.)	4.97 (2.00)	6.33 (2.14)	6.86 (2.20)	7.42 (2.56)	8.71 (1.84)

ANOVA: *p* < 0.0001					

Post hoc analysis among scores: *p* value					

Healing months	≤3	4	5	6	>6
≤3	1				
4	0.0379	1			
5	0.0007	0.6341	1		
6	0.0001	0.0401	0.5711	1	
>6	0.0001	0.0001	0.0001	0.0038	1

**Table 5:** Sensitivity, specificity, and predictive value of the score to identify fracture healing in more than 6 months.

Score	Sensitivity (%)	Specificity (%)	Positive predictive value (%)
3	100	5	27
4	98	12	28
5	94	28	31
6	94	50	38
7	80	65	43
8	63	81	53
9	53	90	65
10	43	97	82
11	20	100	94
12	16	100	100
